# Three-Dimensional-Printed Composite Structures: The Effect of LSCF Slurry Solid Loading, Binder, and Direct-Write Process Parameters

**DOI:** 10.3390/ma17122822

**Published:** 2024-06-10

**Authors:** Man Yang, Santosh Kumar Parupelli, Zhigang Xu, Salil Desai

**Affiliations:** 1 Industrial and Systems Engineering, North Carolina A & T State University, Greensboro, NC 27411, USA; amandayang2005@gmail.com (M.Y.); sparupel@ncat.edu (S.K.P.); 2 Center of Excellence in Product Design and Advanced Manufacturing, North Carolina A & T State University, Greensboro, NC 27411, USA; 3 Mechanical Engineering, North Carolina A & T State University, Greensboro, NC 27411, USA; zhigang@ncat.edu

**Keywords:** 3D printing, binder, ceramic powders, slurry loading, solid oxide fuel cells

## Abstract

In this research, a direct-write 3D-printing method was utilized for the fabrication of inter-digitized solid oxide fuel cells (SOFCs) using ceramic materials. The cathode electrode was fabricated using the LSCF (La_0.6_Sr_0.2_Fe_0.8_Co_0.2_O_3-δ_) slurry loading and the Polyvinyl butyral (PVB) binder. The rheological parameters of slurries with varying LSCF slurry loading and PVB binder concentration were evaluated to determine their effect on the cathode trace performance in terms of microstructure, size, and resistance. Additionally, the dimensional shrinkage of LSCF lines after sintering was investigated to realize their influence on cathode line width and height. Moreover, the effect of the direct-write process parameters such as pressure, distance between the nozzle and substrate, and speed on the cathode line dimensions and resistance was evaluated. LSCF slurry with 50% solid loading, 12% binder, and 0.2% dispersant concentration was determined to be the optimal value for the fabrication of SOFCs using the direct-write method. The direct-write process parameters, in addition to the binder and LSCF slurry concentration ratios, had a considerable impact on the microstructure of cathode lines. Based on ANOVA findings, pressure and distance had significant effects on the cathode electrode resistance. An increase in the distance between the nozzle and substrate, speed, or extrusion pressure of the direct writing process increased the resistance of the cathode lines. These findings add to the ongoing effort to refine SOFC fabrication techniques, opening the avenues for advanced performance and efficiency of SOFCs in energy applications.

## 1. Introduction

The fabrication of solid oxide fuel cells (SOFC) [1,2,3,4] has traditionally involved modifications to both thick- and thin-film manufacturing methods. Multiple layers are assembled and then fired at temperatures between 1300 and 1700 degrees Celsius in thick film processes such as tape casting [5], tape calendaring [6], screen printing [7], and wet spraying [8]. Chemical and physical thin-film deposition techniques including chemical vapor deposition (CVD) [9,10], plasma spraying (PS) [11,12], and spray pyrolysis [13,14] have been utilized to create cell components. For fine local control of chemical composition and/or structure, traditional approaches are not very suitable. Investigating different fabrication techniques is therefore necessary. The 3D printing of SOFC cathode deposition enables numerous advantages compared to traditional manufacturing methods such as chemical vapor deposition (CVD), plasma spraying (PS), and spray pyrolysis. These conventional methods are not particularly well suited for precise local control of chemical composition and/or structure. Thus, there is a need to explore alternative fabrication methods. The flexibility in design and the ability to create complex geometries are major advantages; these make it possible to fabricate microstructures of cathodes with improved performance. The chemical composition and structure of a SOFC can be precisely engineered by depositing the material at desired locations. Besides, the 3D printing of SOFC offers optimization in microstructure characteristics—through the optimization of porosity and surface area—for better electrochemical performance [15]. Conversely, there are some shortcomings such as material compatibility and post-processing steps. Even though 3D-printed cathodes are well suited for the fabrication of SOFC cathode prototypes and small-scale applications, they require inspection and testing to determine their overall performance [16,17]. Overall, 3D printing advances substantial potential for SOFC cathode fabrication, but challenges such as material limitations, process reliability, and cost need to be tackled for broader applications. The research described here employs 3D direct-write printing to allow the chemical composition and structure of a SOFC to be precisely engineered. This allows one to locally control the size and shape of pores in the anode [18,19] and cathode layers, and hence the resulting porosity. The method used for this research is a form of additive manufacturing that utilizes micro-dispensing technology. This method allows the control of the material deposition rate and path simultaneously. This is a major advantage of this process over various more traditional fabrication processes. The direct-write technique is also capable of grading porosity/composition within a layer in addition to grading porosity in successive layers. Ceramic powders [20,21,22,23] are intriguing to control due to their distinct particle nature and inherent lack of cohesion. Organic polymers are often added to improve their moldability. The polymers’ lengthy molecular chains cause liquids to thicken when they dissolve. Because of their stronger intermolecular bonds, polymers with a higher molecular weight produce more viscous solutions. Polyvinyl butyral (PVB) is a binder that is frequently used in ceramic processing to provide slurries with more viscosity and durability. In ceramics, binders are the materials that remain in the body of the pottery after it has dried. The development of porous ceramics necessitates carefully thought-out removal procedures as well as binder choice. The cathode material for single-chamber solid oxide fuel cells [24,25] must possess a few essential characteristics, including electrical conductivity, stability, and porosity. The suggested material for this usage is LSCF (La_0.6_Sr_0.2_Fe_0.8_Co_0.2_O_3-δ_) [26]. In 3D-printing direct-writing methods [27,28,29], the slurries of LSCF powder, a dispersion, and a binder mixed with a solvent are used. This mixture is then printed using a pre-designed pattern design onto a substrate; for the current research investigation, an inter-digitized pattern was selected.

Researchers are exploring numerous microfabrication techniques [30,31,32] to fabricate µ-SOFCs with enhanced efficiency [15,33,34,35,36,37]. Kim et al. demonstrated the robot-dispensing direct-writing technique successfully for the construction of a multilayered planar solid oxide fuel cell (IP-SOFC). The apparatus had three distinct layers: a 20 µm thick cathode, a 15 µm thick electrolyte layer, and a 30 µm thick anode. An open-circuit voltage (OCV) of 1.82 V and a maximum power density of 35 mW/cm^2^ was shown by the manufactured IP-SOFC [36]. A simple and affordable 3D-printing (3DP) technique for creating scalable tubular protonic ceramic fuel cells (PCFCs) was reported by Zoq et al. A dense BCZY27 electrolyte thin film, a porous BCFZY0.1 cathode thin film, and a tubular BCZY27–NiO anode support comprised the painstakingly regulated microstructure of the fabricated single tubular PCFC [38]. Wei et al. investigated making dense 8YSZ electrolytes for solid oxide fuel cells (SOFCs) with a 3D-printing method based on digital light processing (DLP) stereolithography. By employing this technique, the 8YSZ electrolytes were prepared and combined with silver-Ce_0.8_Gd_0.2_O_1.9_ (Ag-GDC) for the cathode and anode, resulting in a symmetric cell arrangement: Ag-GDC|YSZ|Ag-GDC and proficient output functionality [39]. With the use of direct-writing 3D-printing technology, Zhang et al. presented a novel method for creating patterned anode substrates for solid oxide fuel cells. With a maximum power density of 619.44 mW/cm^2^ at 850 °C, the cells built using these anode substrates demonstrated an open circuit voltage of 1.02 V. These results compare favorably to other cell types produced through conventional and non-traditional 3D-printing methods [40]. Rath et al. demonstrated a direct-ink-writing (DIW) printer to build the anode (NiO-ScSZ) and cathode (LSM) of a large-area solid oxide fuel cell (SOFC), with a surface area of 5 × 5 cm^2^. It is underlined that this DIW printing technology could be an affordable way to produce large-area SOFCs with high efficiency and commercial quality. By adding a hybrid-ScSZ layer via magnetron sputtering, this research substantially improved the cell’s performance, long-term stability, and thermomechanical endurance, demonstrating a revolutionary method in SOFC manufacturing [41]. Kuhn et al. fabricated single chamber µSOFCs with interdigitated electrodes consisting of a few 100 microns in size. Results of the study reported that the cells exhibited a peak power density of ~1 mW/cm^2^ and an open circuit voltage of 800 mV. Furthermore, the consequences of these discoveries regarding the possible application of these gadgets in the production of portable electricity were explored [31]. Chhetri et al. created structures for solid oxide fuel cells (SOFC) with customized porosity and microstructure. The goal of the research was to use these novel printing techniques to accurately manage the porosity in the anode and cathode structures [42]. Despite background research in 3D-printed [43,44,45,46] fuel cells, the rheological characterization and process parameter optimization warrant a detailed investigation. Thus, providing processing guidelines to researchers and manufacturers for the consistent fabrication of fuel cells. In our previous work [47,48], we have focused on assessing the deposition characteristics of anode electrodes, which is a comprehensive research, and thus, the current paper focuses on cathode electrodes. Once all the components are optimized, we plan to test the entire fuel cell for its efficiency.

In this research, the rheological characteristics of slurries with different LSCF slurry loading and binder concentration on the properties of the cathode such as microstructure, dimensions, and electrical resistance were evaluated. The dimensional shrinkage of the cathode lines was also evaluated to understand the effect of sintering on SOFC cathode line width and height. In this research, the direct-writing method was employed for the fabrication of the required microstructure for cathode electrode lines, with control over numerous process parameters. These parameters include pressure, distance between nozzle and substrate, slurry mixture ratio, and velocity. Moreover, the microstructure of electrode pattern lines was significantly influenced not only by binder and slurry concentration ratios but also by direct-write process parameters as mentioned above. The analysis of variance was conducted to understand the significant effect of direct-write process parameters on the fabrication of SOFC cathode line traces.

## 2. Methodology

### 2.1. Materials

The cathode electrolyte of SOFCs has been fabricated with LSCF slurries due to their enhanced electrochemical performance [47,48]. LSCF is an efficient electrocatalyst for enhancing surface activity and stability of SOFCs [48]. Other researchers have extensively used lanthanum strontium cobalt ferrite (LSCF) as a staple material for fabricating cathode electrodes for SOFCs due to their thermo-chemical stability in reducing environments, especially during high-temperature operation (600–800 °C) [49]. LSCF has excellent ionic-electronic conducting characteristics for oxygen reduction reactions across the entire cathode cross-section, improving the efficiency of the SOFC [50]. The cathode made use of the LSCF powder attained from the Fuel Cell Material Company, Danbury, CT, USA, which had a 6.3 m^2^/g powder surface area. The cathode slurry was formulated by blending the LSCF particles with the organic solvent terpineol, the binder PVB, and the dispersion Triton X-100. Based on the prior reported experimentation, the binder concentrations of 12% and 15% resulted in porous lines without cracks [51]. A slurry with 60% solid loading has a higher viscosity than those with 40% and 50% solid loading. This study contemplated slurries with three different solid loadings (40%, 50%, and 60%) and two binder concentrations (12% and 15%). Table 1 illustrates the candidate slurry compositions.

### 2.2. Experimental Protocol

**Figure 1:**
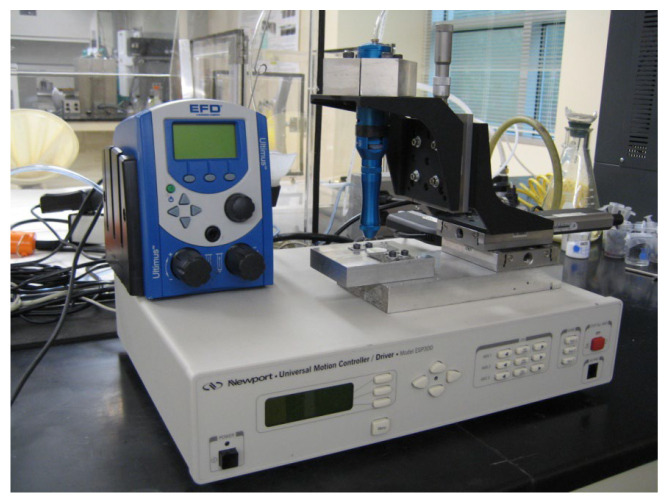
Direct-write 3D-printing system.

The schematic of the direct-write 3D-printing system is illustrated in Figure 1. A 100 µm nozzle was utilized for extruding the cathode slurry, which needed adjustments to the nominal push-out pressures based on solid loading. For the 60% solid loading slurry, the minimum extrusion pressure required was 200 kPa; nonetheless, for slurries with 40% and 50% solid loading, it was about 30 kPa and 100 kPa, respectively. The spacing between the nozzle and substrate throughout direct writing was set at 100 µm at a speed of 0.5 mm/s. Four cathode lines were printed on the YSZ substrate under every pressure. The cathode lines before and after sintering were examined by a Zeiss microscope, and four data points were recorded per slurry. The cathode lines were captured in both top and profile views. The Image-pro plus software, V.10 was utilized to determine the height and width of the lines before and after sintering. Moreover, the conductivity of the lines was measured using a four-point probe. The micro-solid oxide fuel cell’s performance can be significantly impacted by the cathode resistance. The cathode line microstructure can be impacted by composition, substrate surface qualities, and the sintering process of the slurry. A Signatone Pro4 and a Keithley 2400 series source meter from Signatone Corporation, Gilroy, CA, USA were employed to determine the resistance of sintered lines. The resistance of cathode traces was measured at 20 °C to maintain consistency in the measurements across different samples.

The parameters considered while investigating the direct-write process included nozzle speed, distance between the nozzle and substrate, and slurry chamber pressure. Substrates with constant surface preparation were prepared. The optimal values for the solid loading, binder, and dispersant were chosen based on the prior and this research work [48,51,52,53,54]. The factorial experimental design is illustrated in Table 2, which includes three components and two levels each.

## 3. Results

### 3.1. Effect on Slurry Rheology

**Figure 2:**
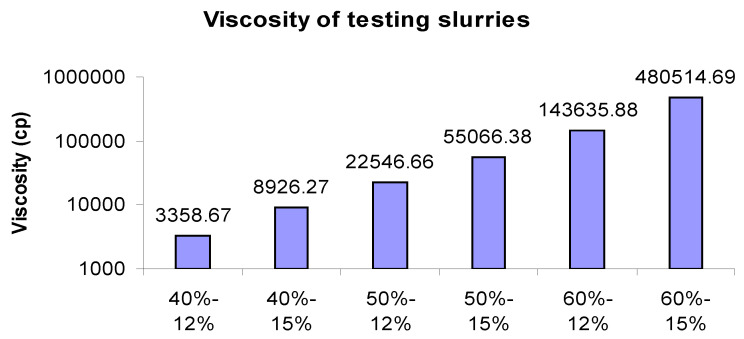
Viscosities of test slurries.

The viscosities of various combinations were measured at 0.08 s^−1^ shear rate as illustrated in Figure 2. The slurry viscosity increased significantly with the increase in the solid loading or binder concentrations. Between these two factors, solid loading has a major impact, as solid loading with a 20% increase led to a further increase in viscosity compared to the 25% increase in binder concentration. All slurries with lower solid loading had lower viscosity than those with higher solid loading, as can be seen in Figure 2.

**Figure 3:**
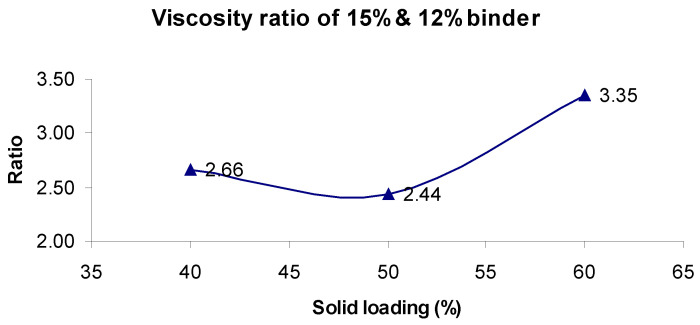
Solid loading vs. viscosity ratio of 15% and 12% binder concentration.

Figure 3 illustrates that the viscosity of the slurry increased by 25% for different solid loading when the binder concentration was varied from 12% to 15%. The 40% and 50% solid loadings slurries exhibited more than twofold increase in their viscosities, whereas the 60% solid loading slurry had a nearly 335% increase in the slurry viscosity. The addition of altered concentrations of binder media resulted in modifying both the fluid viscosity and its rheological behavior. An increase in the binder concentration from 12 to 15% resulted in higher viscosities for each solid loading of the slurry. The 40% solid loading slurry observed 2.66-fold increase in viscosity, whereas a 50% solid loading slurry observed 2.44-fold increase in viscosity. This can be attributed to the higher particle/particle interactions of higher binder concentrations, which tends to increase the effective rheological resistance [51,53].

**Figure 4:**
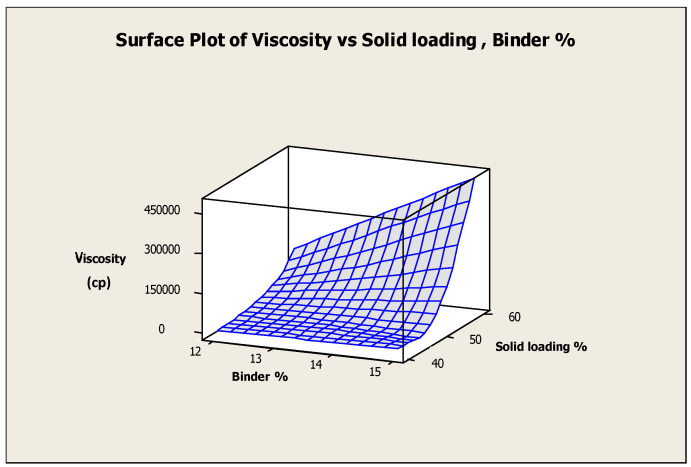
Surface plot of viscosity vs. solid loading and binder.

The surface plot of viscosity vs. solid loading and binder concentrations was illustrated in Figure 4 to estimate trend patterns. Slurries with low solid loading and binder concentrations had decreased viscosity values, and vice versa. The effect of solid loading, binder concentrations, and their interaction effects on the slurry viscosity was evaluated by conducting an ANOVA analysis. From the ANOVA results as shown in Table 3, it can be concluded that the solid loading, binder concentrations, and their interaction effects are statistically substantial for the viscosity of slurries. Nevertheless, solid loading has a superior impact on the slurry viscosity.

### 3.2. Effect of Solid Loading and Binder Concentration on Dimensions of LSCF Lines

**Figure 5:**
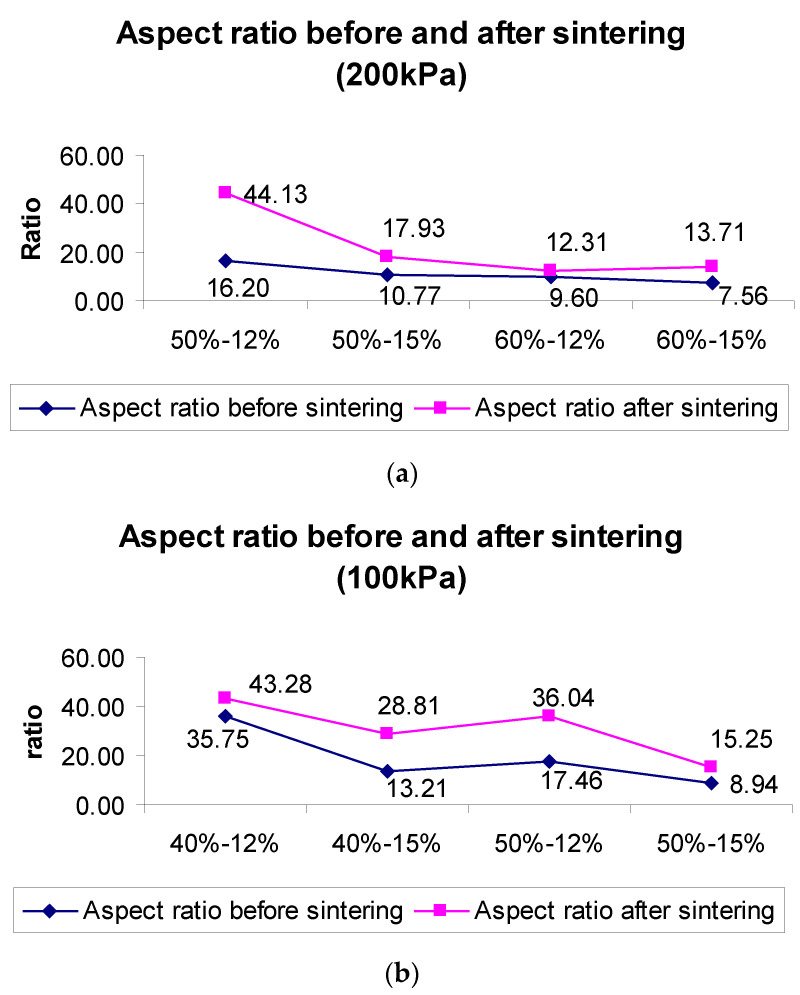
(**a**) Aspect ratio before and after sintering under 200 kPa, (**b**) Aspect ratio before and after sintering under 100 kPa.

The dimensions of the LSCF lines were evaluated using the direct-write process. Slurries based on different solid loadings were extruded under 200 kPa, 100 kPa, and 30 kPa, respectively. It was observed that with the increase in the binder and solid loading concentrations, the width of the line was reduced. However, an increase of 20% in solid loading resulted in a higher width reduction than that affected by an increase of 25% in binder concentration. Thus, it can be inferred that solid loading concentrations affect the direct-write line’s width more drastically. The aspect ratio of the line was employed in this work to determine the dimensional characteristics of the cathode lines. In this instance, the aspect ratio equals width over height. Figure 5a,b demonstrate the aspect ratio under 200 kPa and 100 kPa, respectively. The rheological properties of the slurry influenced the aspect ratio. An increase in the slurry viscosity leads to a lower aspect ratio and vice versa. The aspect ratio decreased with the increase in the solid loading and binder concentrations. This can be attributed to the fact that for a slurry with higher binder concentration, and solid loading has a higher viscosity which further influences the aspect ratio. Thus, higher viscosities resulted in a lower spread of the slurries on the electrolyte substrate to form the cathode trace. The lower spread directly correlates with the lower width of the cathode traces and thereby, a reduction in the aspect ratio (width/height ratio). Figure 5 clearly shows that the highest solid loading slurry at 60% had the lowest aspect ratio, followed by 50% solid loading at 200 kPa extrusion pressure. The 60% slurry loading could not be extruded at 100 kPa due to high viscosity. Similarly, at 100 kPa extrusion pressure, the 50% solid loading slurry had a lower spread and subsequent width as compared to the 40% solid loading slurry. Also, it is important to note that an increase in binder concentration from 12% to 15% in each of the slurries resulted in higher viscosity and thereby, a subsequent reduction in aspect ratio.

### 3.3. Evaluation of Dimensional Shrinkage of the LSCF Lines

**Figure 6:**
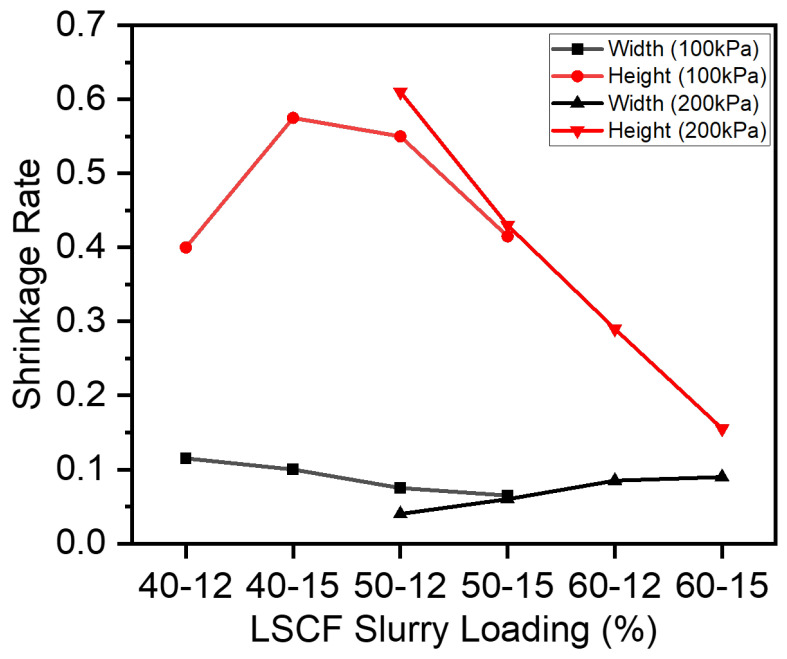
Shrinkage of different solid loading slurries under varying extrusion pressures.

The binder additive and solvent decompose during the sintering process step, leading to a shrinkage in the dimensions of the cathode line width and height. The additives, which include Triton dispersant and PVB binder, and solvents, such as α-terpineol decomposition and removal, are complex processes. The resistance of the cathode lines can be impacted by the line’s width and height. To determine how to control the LSCF lines’ shrinkage, it is very crucial to understand the effect of solid loading and the binder’s concentrations on the shrinkage of slurries. The width and height shrinkage of 40% and 50% solid loading slurries under 100 kPa is illustrated in Figure 6. Except for the slurry with 40% solid loading and 12% binder, with increasing solid loading and binder concentration, the width shrinkage decreased. This can be attributed to higher viscosities of higher solid-loading slurries. A higher viscosity slurry creates interparticulate resistance and thereby, the cathode trace deposited on the electrolyte substrate has a smaller footprint. Subsequently, the shrinkage in smaller dimensions (width and height) will be much lower as compared to less viscous slurries which tend to spread out wider on the electrolyte substrate. Thus, the 40% solid loading slurry had relatively higher shrinkage rates. Moreover, higher solid-loading slurries cannot be extruded at 100 kPa and need higher extrusion pressures (200 kPa). The width and height shrinkage of 50% and 60% solid loading slurries under 200 kPa is also illustrated in Figure 6. With the increase in solid loading and binder concentration, the shrinkage of width increased. Nonetheless, the shrinkage in height decreased when the binder concentration increased.

**Figure 7:**
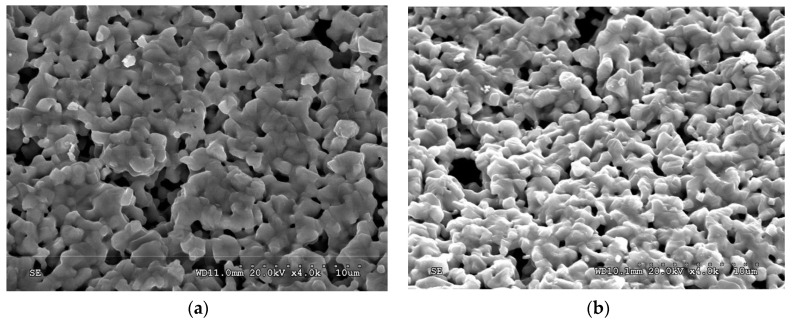
SEM images of the SOFC cathode trace (**a**) 50% solid loading–12% binder and 0.2% dispersant, (**b**) 50% solid loading–15% binder and 0.2% dispersant.

The porosity of the cathode lines plays a vital role in the performance of the SOFC as it has a major impact on the gas permeability inside the SOFC. Scanning electron microscopy (SEM) was utilized to analyze the porosity of the SOFC cathode lines fabricated with different binder concentrations as illustrated in Figure 7. The SEM images for different slurry depositions were evaluated using the Image J, Version 1.54i [55] image processing software. Each SEM image was converted to a RGB format and then threshold values were set to demarcate the porous areas from the rest of the image. Percentage porosity values were recorded for different samples. Denser SOFC cathode microstructures had porosity values ranging from 8% to 12% whereas, sparser SOFC cathodes had higher porosity ranging from 18% to 25%. These porosity values were correlated with resistance measurements of the traces based on the connected microstructure network. The results of the SEM analysis illustrated that the increase in the binder concentration led to an increase in the porosity. The findings report that a fine control over the porosity was achieved by varying the binder concentration as shown in Figure 7a,b.

**Figure 8:**
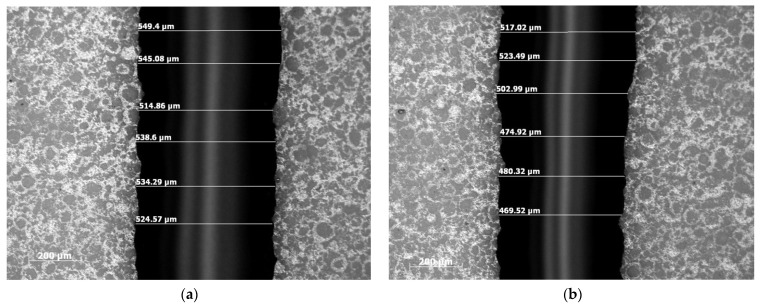
Optical microscope images of the SOFC cathode line (width) with 40% solid loading–12% binder and 0.2% dispersant (**a**) before sintering (**b**) after sintering.

**Figure 9:**
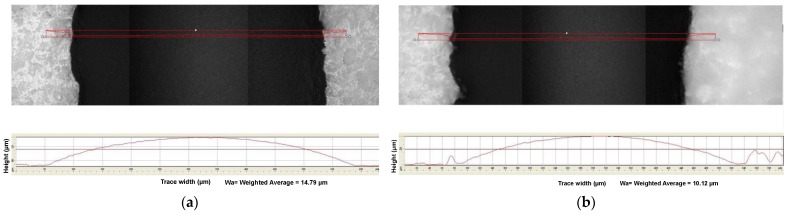
Surface profilometric images of the SOFC cathode line (height) with 60% solid loading-12% binder and 0.2% dispersant (**a**) before sintering (**b**) after sintering.

The shrinkage in the width and height of the cathode line was analyzed using the optical microscope and surface profilometer, respectively, for various concentrations of LSCF slurry loading and binder concentrations. Figure 8 illustrates the SOFC cathode line microscope images before and after sintering for 40% solid loading, 12% binder, and 0.2% dispersant. The cathode lines exhibited a shrinkage ratio of 0.11 in terms of the width before and after sintering as shown in Figure 8a,b, whereas the height shrinkage of the cathode line with 60% solid loading, 12% binder, and 0.2% dispersant exhibited a shrinkage rate of 0.30 as shown in Figure 9a,b before and after sintering, respectively. It is very crucial to understand the effect of solid loading and binder concentrations on the shrinkage of cathode lines. Thus, further analysis was performed in the following section to analyze their impact on the height and width shrinkage. 

A design of experiments and analysis was conducted to understand the relationship between shrinkage, solid loading, and binder concentration. The analysis of variance (ANOVA) results for width shrinkage (200 kPa) are presented in Table 4. For the width shrinkage, all the main and interaction effects are statistically significant. The most significant factor was determined to be the binder concentration. As reported in a previous research study, the slurry viscosity is dependent on the solid loading concentration.

Due to the shrinkage of the line dimensions, the surface and interfacial tension occur. The compositions of the slurries impact these two tensions which include surface and interfacial tension and their ratio directly. Generally, while sintering the organic components and solvent, the slurry with lower viscosity will possess larger shrinkage. ANOVA results illustrate that the binder concentration plays a crucial role in width shrinkage. This can be justified as the binder’s long molecular chains strengthen the intermolecular forces resulting in higher interfacial tension and surface tension. Table 5 shows the ANOVA results for height shrinkage under 200 kPa. At a 95% significance level, both the solid loading and binder impact the height shrinkage.

Both the width and height shrinkage increased significantly when the solid loading concentration was increased. Likewise, when the binder concentration was increased, the height shrinkage was increased, and the width shrinkage was reduced. This can be rationalized by the fact that the slurry can be extruded more easily for low-viscosity slurries compared to high-viscosity ones. This results in a higher aspect ratio. Therefore, the interfacial tension plays an influential role in the width and height shrinkage.

### 3.4. Evaluation of Electrical Resistance of the Cathode Lines 

**Figure 10:**
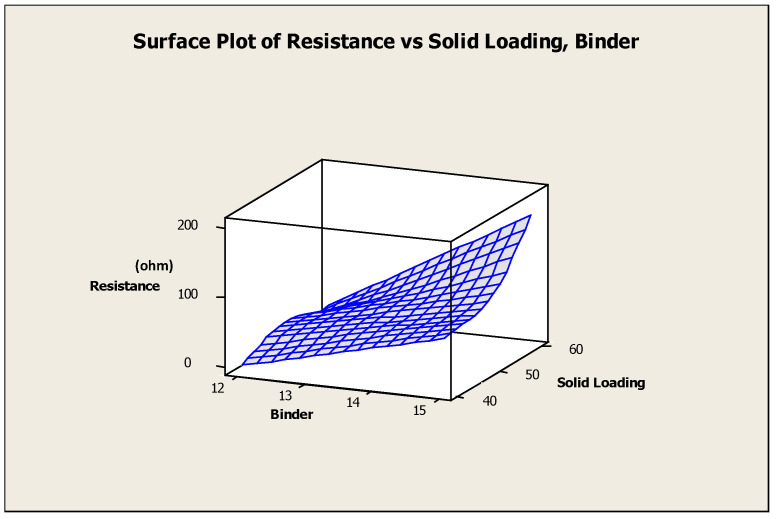
Surface plot of resistance vs. solid loading and binder.

Generally, the cathode line resistance is dependent on the line’s microstructure, the slurry’s solid loading, and cross-section dimensions. In this section, these factors were evaluated. Figure 10 illustrates a plot of resistance vs. solid loading and binder concentrations which evaluates the trend patterns. Lower values of resistance were obtained for slurries with lower binder concentration and vice versa. The cathode lines directly written with 15% binder and 60% solid loading concentration had the highest resistance, and the cathode line with 12% binder, and 40% solid loading had the lowest resistance. The cathode line directly written by 50% solid loading had a relatively higher resistance than that written by 60% solid loading at lower binder concentrations. Nonetheless, the resistance increased with solid loading concentrations for the higher binder concentrations.

The effect of solid loading and binder concentrations on the resistance of the extruded cathode lines at 200 kPa and 100 kPa extrusion pressures, respectively, was evaluated with ANOVA. The resistance of the cathode lines was significantly impacted by both binder and solid loading factors. The resistance increased with the increase in the concentration of the binder. A higher porosity of the microstructure resulted due to the higher binder concentration. This resulted in decreased connectivity of the LSFC material, thereby increasing the cathode line resistance.

### 3.5. Data Collection and Statistical Analysis

A full factorial design was employed in this research to evaluate the output responses, which included line width, height, and electrical resistance. Table 6 shows output responses for the slurry with 50% solid loading, 12% binder, and 0.2% dispersant concentrations, respectively. For the combination experiment, no. 5 was the worst-case scenario for the 3D printing of LSCF trace for the optimal composition of 50% solid loading, 12% binder, and 0.2% dispersant. This is because the nozzle was at the farthest point from the substrate (200 µm), fastest speed (1 m/s), and lowest extrusion pressure (100 kPa). These are the extreme conditions, where the slurry would not be extruded from the nozzle and if it did extrude, it would not deposit as a continuous line trace. Thus, no cathode trace was deposited at this setting. For the combination (exp. no. 2): distance = 200 µm, speed = 0.5 mm/s, and pressure = 100 kPa, a minimal resistance of around 3.6–5.6 KΩ was attained.

**Figure 11:**
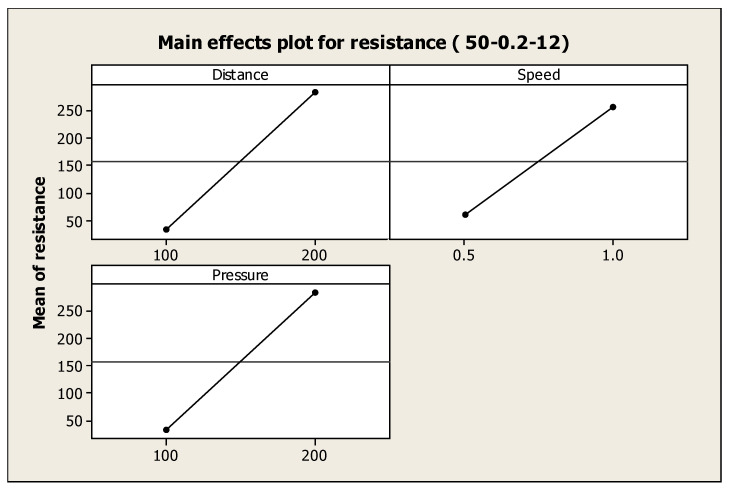
Main effects plot for resistance (50-0.2-12).

The 50% solid loading slurry was the ideal slurry concentration for investigation as it had the least resistance among all slurries examined. Table 7 demonstrates that the main effects of distance, pressure, and their interaction were statistically significant with respect to resistance. Figure 11 illustrates that an increase in the speed, distance, and pressure of the direct writing process increases the cathode line trace resistance.

The ANOVA results for the effect of different parameters such as distance, speed, pressures, and their interactions on the width and height of the line after sintering, respectively were presented in Table 8 and Table 9. The results indicate that both the main and interaction effects are significant for the width and height after sintering. However, the interaction between pressure and speed was not significant for width after sintering, and the interaction between pressure and distance was not significant for height after sintering. 

### 3.6. Evaluation of Extrusion Pressure, Line Dimensions and Resistance

All three parameters are significant for the line dimensions. However, distance and pressure are significant for the cathode line resistance. Hence, in this section, the cathode line dimension, the extrusion pressure, and resistance were investigated. When all other direct-writing parameters are kept constant, the width of the cathode line trace was dependent on the extrusion pressure. A regression analysis was performed to predict the width of the cathode line before sintering. A linear relationship was observed between the pressure and width before sintering. The regression analysis of width before sintering versus pressure is presented in Table 10. Further, to predict the width of the line after sintering, a regression analysis was conducted. The regression analysis of width after sintering versus pressure is illustrated in Table 11. The width (µm) = 393.25 + 0.99 pressure (kPa) before the sintering scenario, for after the sintering scenario the width (µm) = 358.28 + 0.96 pressure (kPa).

From the above findings, it can be inferred that the microstructure of SOFC cathode line traces is significantly impacted by the extrusion pressure, nozzle speed, and the distance between the nozzle and substrate. ANOVA results illustrate that all three direct-write extrusion parameters are significant for the line electrical resistance. An increase in the distance, speed, or pressure of the direct writing process increases the electrical resistance. The parameters are insignificant for height and width shrinkage. This work uses 3D direct-write printing to precisely engineer the structure and chemical makeup of a solid oxide fuel cell. As a result, the cathode layers’ can be locally controlled for size and form, and the resulting optimal porosity.

Gardner et al. illustrated the fabrication of SOFC cathode by utilizing the aerosol jet printing technique. The cathode and buffer layers were printed from La_0.6_Sr_0.4_Co_0.2_Fe_0.8_O_3−x_ (LSCF), and gadolinium-doped ceria (Ce_0.9_Gd_0.1_)O_1.95_ (CGO) inks, respectively. The results illustrated a 19% improvement in the current density using LSCF slurry paste compared to La_1−x_Sr_x_MnO_3_ (LSM) material. However, further fine-tuning of aerosol jet printing parameters is needed to control the microstructure of the cathode fuel cell. Moreover, aerosol jet printing has limited material compatibility, lower resolution, and inferior surface roughness [56]. Qiang et.al demonstrated the fabrication of A-site-deficient La_0.58_Sr_0.4_Co_0.2_Fe_0.8_O_3−δ_ cathode and PVB binder for SOFC by a screen-printing method. The study reports that the PVB binders enhanced L58SCF cathode performance by yielding uniform microstructure and crack-free surface. The endurance analysis of the SOFCs with the candidate cathode—L58SCF slurry paste needs to be determined to understand the long-term electrochemical behavior of the fuel cell. Additionally, the impact of current transfer and gas diffusion because of the variations in the cathode thickness needs to be investigated to study their impact on the performance of the SOFCs [57]. Anelli et al. proposed a novel hybrid 3D-printing process that includes inkjet printing and robocasting for the fabrication of symmetrical electrode-supported solid oxide cells. Their research group studied the rheological characteristics and the printability evaluations of the robocasting LSM-YSZ slurries for the electrodes and inkjet printing using water-based YSZ ink for the dense electrolyte. However, the robocasting and ink-jet methods are extremely laborious and complex processes to create micro-nanoscale patterns and bound to lab-based proof-of-concept solutions [58]. Rath et al. demonstrated the fabrication of durable and highly efficient SOFC using Nio-ScSz for anode and LSM for cathode by utilizing the direct-ink-write printing method. The uniform microstructure of the electrodes was achieved by optimizing the viscosity of the cathode and anode ink materials. Further, the magnetron sputtering method was employed to enhance the electrochemical performance of the 3D-printed cell by 21%. The viscosity of the anode and cathode inks plays a crucial role in the fabrication of the SOFCs thus, further analysis needs to be conducted on various ink formulation concentrations of the Nio-ScSz and LSM for enhanced printability and cell performance [41].

Our research in contract focuses on direct-write 3D-printing methods for the fabrication of inter-digitized solid oxide fuel cells (SOFCs) using ceramic materials [59]. This research work focuses on understanding the effect of LSCF slurry solid loading, binder, and direct-write process parameters for fabricating µ-SC-SOFCs using 3D printing. The LSCF slurries have substantially varying viscosities and fluid rheology behavior, and thus it is critical to assess the optimal combination of dispersants, binder, and solid loading to enable effective 3D printing. Moreover, the direct-write process parameters were investigated to determine their significant impact on the microstructure of cathode line fabrication. The detailed analysis of the slurry rheology of the various concentrations of the slurry loading and binder concentration was investigated. Moreover, to our knowledge, there is very limited research work conducted in the literature using the direct-write 3D-printing method for the fabrication of SOFCs. The direct-write method implemented in this research enables the fabrication of SOFC cathode lines by optimizing the concentrations of the slurry loading and binder. Further, the direct write process parameters which include nozzle speed, nozzle size, distance between the nozzle and substrate, and extrusion pressure were optimized to enhance the electrical resistance of the cathode lines. These findings add to the continuing effort to refine SOFC manufacturing techniques in energy conversion and several industrial applications.

## 4. Conclusions

In this research, a direct-write 3D-printing technique was demonstrated for the fabrication of solid oxide fuel cell cathode line traces. With this technique, it is possible to concurrently regulate the path and rate of material deposition. The effects of solid loading and binder concentrations on the cathode line dimensions were estimated to observe their effect on the cathode line dimension, microstructure, and electrical resistance. LSCF solid loading and binder concentrations not only affected the slurry’s viscosity but also affected the slurry’s rheology behavior. From this study, it can be reported that LSCF solid loading plays a significant role in both rheology behavior and line dimension. Further, aspect ratio and dimensional shrinkage were studied. The highest electrical resistance occurred when the width after being sintered was low but the height after being sintered was high, whereas the lowest resistances occurred when the height after the sinter was low and the width after the sinter was high. Various LSCF slurry compositions were examined to determine the optimal slurry that can generate a lower line width and lower electrical resistance. Based on the research findings, a slurry with 50% solid loading, 12% binder, and 0.2% dispersant was preferred to conduct direct writing parameters research analysis. The microstructure of cathode electrode pattern lines is not only affected by the composition of the slurry but also by the direct-writing process parameters. These process parameters include nozzle speed, nozzle size, distance between the nozzle and substrate, extrusion pressure, and the diffusion rate of the electrolyte substrate. The direct-write process parameters were evaluated by a 2^3^ (k = 3) full factorial experimental design. Further, a statistical analysis was utilized to determine their effects on resistance, dimension, and shrinkage of dimension. Based on an ANOVA finding, pressure, and distance had significant effects on the cathode electrode resistance. An increase in the distance between the nozzle and substrate, speed, or extrusion pressure of the direct writing process increased the resistance of the cathode lines. These findings add to the continuing effort to refine SOFC manufacturing techniques, opening the path for enhanced performance and efficiency in energy conversion applications.

## Figures and Tables

**Table 1:** Candidate slurry compositions.

Sample Number	Solid Loading (%)	Dispersant (%)	Binder (%)
1	60	0.2	12
2	60	0.2	15
3	50	0.2	12
4	50	0.2	15
5	40	0.2	12
6	40	0.2	15

**Table 2:** Factor level settings.

Level	Pressure	Distance	Speed
Low (−)	100 kPa (for 50%) 200 kPa (for 60%)	100 µm	0.5 mm/s
High (+)	200 kPa (for 50%) 300 kPa (for 60%)	200 µm	1 mm/s

**Table 3:** ANOVA table for solid loading, binder, and viscosity.

Source	DF	Seq SS	Adj SS	Adj MS	F	*p*
Solid Loading	2	2.24 × 10^11^	2.24 × 10^11^	1.12 × 10^11^	3.18 × 10^4^	0.00
Binder	1	4.78 × 10^10^	4.78 × 10^10^	4.78 × 10^10^	1.35 × 10^4^	0.00
Solid loading*Binder	2	6.93 × 10^10^	6.93 × 10^10^	3.46 × 10^10^	9.84 × 10^3^	0.00
Error	6	2.11 × 10^7^	2.11 × 10^7^	3.52 × 10^6^		
Total	11	3.41 × 10^11^				
S = 1875.61	R-sq = 99.99%		R-Sq(adj) = 99.99%			

**Table 4:** ANOVA table for solid loading, binder, and width shrinkage (200 kPa).

Source	DF	Seq SS	Adj SS	Adj MS	F	P
Solid Loading	1	0.0162	0.0162	0.0162	43.20	0.0030
Binder	1	0.1104	0.1104	0.1104	294.53	0.0000
Solid loading*Binder	1	0.1152	0.1152	0.1152	307.20	0.0000
Error	4	0.0015	0.0015	0.0004		
Total	7	0.2434				
S = 0.0193649	R-sq = 99.38%		R-Sq(adj) = 98.92%			

**Table 5:** ANOVA table for solid loading, binder, and height shrinkage (200 kPa).

Source	DF	Seq SS	Adj SS	Adj MS	F	*p*
Solid loading	1	0.1770	0.1770	0.1770	59.7500	0.0020
Binder	1	0.0496	0.0496	0.0496	16.7500	0.0150
Solid loading*Binder	1	0.0010	0.0010	0.0010	0.3400	0.5900
Error	4	0.0119	0.0119	0.0030		
Total	7	0.2395				
S = 0.0544289	R-Sq = 95.05%		R-Sq(adj) = 91.34%			

**Table 6:** Output responses for 50% solid loading, 12% binder, and 0.2% dispersant.

No.	Direct Writing Parameters			Before Sintering		After Sintering		Shrinkage of Width	Shrinkage of Height	Resistance (KΩ)
	Distance (µm)	Speed (mm/s)	Pressure (kPa)	Width (µm)	Height (µm)	Width (µm)	Height (µm)			
1	100	0.5	100	503.90	31.5	467.2	25.80	0.07	0.18	22.00
1	100	0.5	100	394.70	11.2	375.5	20.50	0.05	−0.83	101.90
2	200	0.5	100	447.60	19.7	475.5	24.70	−0.06	−0.26	5.68
2	200	0.5	100	441.40	30.5	407.8	23.80	0.08	0.22	3.69
3	100	1.0	100	298.70	35.9	294.6	20.80	0.01	0.42	57.76
3	100	1.0	100	289.70	22.6	283.3	20.50	0.02	0.09	69.45
4	100	0.5	200	614.90	31.8	617.6	27.70	0.00	0.13	22.00
4	100	0.5	200	697.10	34.7	609.7	28.60	0.13	0.17	235.50
5	200	1.0	100	000.0 *	00.0	000.0	00.0 *	1.00	1.00	10,000.0 **
5	200	1.0	100	000.00	00.0	000.0	00.00	1.00	1.00	10,000.00
6	100	1.0	200	444.20	29.4	463.1	27.20	−0.04	0.08	3.15
6	100	1.0	200	407.10	22.9	416.7	28.20	−0.02	−0.23	8.35
7	200	0.5	200	381.90	26.1	363.9	23.90	0.05	0.08	104.70
7	200	0.5	200	379.90	24.6	412.9	24.20	−0.09	0.02	236.70
8	200	1.0	200	348.60	25.7	326.5	16.30	0.06	0.37	1407.00
8	200	1.0	200	353.40	27.7	331.0	16.70	0.06	0.40	498.50

Nomenclature: * that width and height equal 0 denotes that the slurry was not extruded. ** For dotted lines and no lines written, the resistance was shown as 10,000 KΩ.

**Table 7:** ANOVA table for resistance.

Source	DF	Seq SS	Adj SS	Adj MS	F	*p*
Distance	1	248,339	248,339	248,339	4.68	0.06
Speed	1	153,914	153,914	153,914	2.90	0.13
Pressure	1	249,551	249,551	249,551	4.70	0.06
Distance*Speed	1	148,272	148,272	148,272	2.79	0.13
Distance * Pressure	1	383,419	383,419	383,419	7.22	0.03
Speed*Pressure	1	156,298	156,298	156,298	2.94	0.13
Distance*Speed*Pressure	1	153,189	153,189	153,189	2.89	0.13
Error	8	424,671	424,671	53,084		
Total	15	1,917,655				
S = 230.399	R-Sq = 77.85%		R-Sq(adj) = 58.48%			

**Table 8:** ANOVA for width after sintering.

Source	DF	Seq SS	Adj SS	Adj MS	F	*p*
Distance	1	91,502	91,502	91,502	82.44	0.000
Speed	1	163,020	163,020	163,020	146.88	0.000
Pressure	1	95,730	95,730	95,730	86.25	0.000
Distance*Speed	1	9522	9522	9522	8.58	0.019
Distance * Pressure	1	1145	1145	1145	1.03	0.400
Speed*Pressure	1	29,003	29,003	29,003	26.13	0.001
Distance*Speed*Pressure	1	44,803	44,803	44,803	40.37	0.000
Error	8	8879	8879	1110		
Total	15	443,604				
S = 33.3148	R-Sq = 98.0%		R-Sq(adj) = 96.25%			

**Table 9:** ANOVA for height after sintering.

Source	DF	Seq SS	Adj SS	Adj MS	F	*p*
Distance	1	228.98	228.98	228.98	16.69	0.003
Speed	1	227.47	227.47	227.47	16.68	0.004
Pressure	1	272.84	272.84	272.84	20.01	0.002
Distance*Speed	1	280.82	280.82	280.82	20.59	0.002
Distance * Pressure	1	0.05	0.05	0.05	0.00	0.952
Speed*Pressure	1	49.17	49.17	49.17	3.61	0.094
Distance*Speed*Pressure	1	92.89	92.89	92.89	6.81	0.031
Error	8	109.11	109.11	13.64		
Total	15	1261.33				
S = 3.69298	R-Sq = 91.35%		R-Sq(adj) = 83.78%			

**Table 10:** Regression analysis: width (µm) before sintering vs. pressure (kPa).

Predictor	Coef	SE Coef	T	*p*
Constant	393.25	42.15	9.33	0
Pressure (kPa)	0.99	0.11	9.13	0
S = 64.0232	R-sq = 89.30%		R-Sq(adj) = 88.20%	

**Table 11:** Regression Analysis: width (µm) after sintering vs. pressure (kPa).

Predictor	Coef	SE Coef	T	*p*
constant	358.28	34.48	10.39	0
Pressure (kPa)	0.96	0.09	10.79	0
S = 52.3727	R-sq = 92.10%		R-Sq(adj) = 91.30%	

## Data Availability

Data are contained within the article.
